# Laryngeal Vibration to Treat Abductor‐Type Laryngeal Dystonia: Effectiveness and Cortical Response

**DOI:** 10.1002/lary.70020

**Published:** 2025-08-08

**Authors:** Arash Mahnan, Jiapeng Xu, Jinseok Oh, Divya Bhaskaran, Yang Zhang, Peter J. Watson, Jürgen Konczak

**Affiliations:** ^1^ Meta Reality Labs Redmond Washington, DC USA; ^2^ Human Sensorimotor Control Laboratory, School of Kinesiology, University of Minnesota Minneapolis Minnesota USA; ^3^ Center for Clinical Movement Science, University of Minnesota Minneapolis Minnesota USA; ^4^ Department of Speech, Language, and Hearing Sciences University of Minnesota Minneapolis Minnesota USA

**Keywords:** EEG, human, somatosensory, spasmodic dysphonia, speech

## Abstract

**Objectives:**

Demonstrate proof‐of‐concept that superficial vibro‐tactile stimulation (VTS) of the larynx can serve as a non‐invasive neuromodulation method to reduce voice symptoms in people with abductor‐type laryngeal dystonia (ABLD) and record the underlying neural response to VTS of the somatosensory‐motor cortex.

**Methods:**

Using a wearable collar with embedded vibrators, 11 people with ABLD received VTS for 24 min. To assess voice effects, they vocalized vowels and spoke standardized test sentences and words. Cortical activity was recorded using 64‐channel EEG. S*moothed cepstral peak prominence*, cumulative word and sentence duration were derived from voice recordings as objective markers of speech quality next to perceived speech effort as a subjective marker.

**Results:**

In response to VTS, 64% of participants rated their improvement in voice quality as noticeable to very noticeable. Analysis of objective voice measures indicated a reduction in voice symptoms in up to 45% of participants immediately after and/or 20 min past the cessation of VTS. The cortical response to VTS application was a reduced event‐related spectral power in theta, alpha, and beta bands over left and right somatosensory‐motor cortical areas that was most prominent over the left premotor cortex in 7/11 participants.

**Conclusion:**

Applying laryngeal VTS proved to be feasible and safe. VTS can induce acute short‐term reductions in voice symptoms, which is important given the limited therapeutic options for ABLD. The electrocortical correlate of VTS was an event‐related desynchronization of neuronal firing patterns over bilateral somatosensory‐motor cortex.

**Level of Evidence:**

4.

## Graphical abstract

In this study, people with abductor‐type laryngeal dystonia wore a collar with small, embedded vibrators. After vibrating the skin above the Adam's apple for 24 min, two‐thirds of participants thought their voice had noticeably improved, and objective measures of voice documented improvements in 45% of participants. The study shows that vibration of the larynx can temporarily reduce the voice symptoms of people with abductor‐type laryngeal dystonia, which is important because this patient group lacks effective treatments.

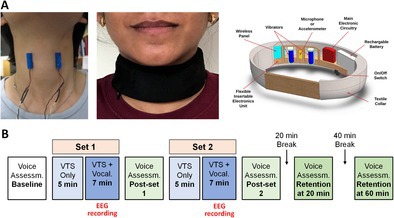

## 1 Introduction

Laryngeal dystonia (LD) is a task‐specific focal dystonia characterized by involuntary spasms of the laryngeal musculature during speech [1, 2]. It is a rare speech disorder affecting up to 35.1 per 100,000 of the general population [3]. LD develops spontaneously during midlife with gradual onset in the first year and then becomes chronic for life. Adductor‐type LD (ADLD) is most common. Approximately 20% of patients present with abductor‐type LD (ABLD), which is characterized by hyperabduction, uncontrolled vocal fold opening resulting in breathy or prolonged speech [1, 4]. A common therapeutic option is Botulinum neurotoxin (BoNT) injection to the laryngeal muscles that provide an average symptom relief of 90% for those with ADLD and 67% for those with ABLD [5, 6]. Yet for ABLD, reaching the target muscle posterior cricoarytenoid is challenging, needle placement may be unsuccessful, and is not well tolerated by all [7], leaving part of this group with limited effective treatment options.

The neuropathology of LD is still incompletely understood, but LD involves structural and functional changes in a network comprising the basal ganglia–thalamo‐cortical circuitry, brainstem, cerebellum, somatosensory and motor cortex [8, 9, 10]. Electrocortical abnormalities in LD include a widespread decrease in cortical inhibition [11] and an abnormally high synchronous activity within and across cortical neural networks involved in voice production, primarily lateralized in the left hemisphere [12].

LD shares somatosensory deficits with other forms of focal dystonia. For example, upper limb proprioceptive deficits were confirmed in LD [13], while patients with cervical dystonia or blepharospasm exhibited elevated tactile discrimination and finger position sense thresholds [14, 15, 16]. Moreover, touching areas of or near the dystonic musculature (i.e., sensory trick) may provide symptom relief [17, 18]. These findings open an avenue for a missing treatment for LD. Specifically, vibro‐tactile stimulation (VTS) of the larynx could be the suitable neuromodulation tool, given that it is known to alter afferent signals from the vibrated mechanoreceptors in muscles and skin [19, 20] and, thus, could stimulate proprioceptive and tactile receptors of the larynx [21]. Recent research from our group investigated the effect of laryngeal VTS on speech quality and cortical activity in ADLD. A single 30‐min VTS session induced almost immediate improvements in speech quality in 69% of participants [22], and an 11‐week longitudinal study with 39 ADLD participants showed that 57% experienced repeated meaningful benefits such as reduced voice effort [23]. This behavioral effect was associated with a suppression of low frequency oscillations over the left somatosensory‐motor cortex, suggesting that VTS can lead to a normalization of excessive synchronous activity of somatosensory‐motor cortical networks underlying LD [22].

This proof‐of‐concept study extends our previous research on laryngeal VTS in ADLD to investigate its potential efficacy for people with ABLD. Given the current lack of effective treatments for ABLD, demonstrating that VTS may alleviate voice symptoms in this population would be a significant advancement. We applied laryngeal VTS in a single session and recorded voice and EEG signals to determine if VTS can reduce voice symptoms and, if so, document the electrocortical signature of such behavioral effect.

## 2 Materials and Methods

### 2.1 Participants

Eleven people diagnosed with abductor‐type LD (10 female; mean age ± std.: 56.1 ± 10.6 years) participated (see Table 1 for clinical characteristics of study participants). Participants were recruited through the Dysphonia International organization and had been diagnosed with ABLD by a trained speech‐language pathologist or otolaryngologist. The average disease duration was 11.5 ± 5.8 years. Two participants presented with voice tremor (S02, S09). Three participants had never received BoNT. For seven participants who had previously received injections, the average time since the last BoNT injection was 7.5 ± 5.5 years. They had discontinued BoNT treatment due to a lack of effectiveness and did not report any other ongoing effective treatment. One participant (S10) received BoNT injections every 3 months. At the time of testing, S10 was 85 days past her last injection and symptomatic during assessment. All participants provided informed consent prior to the experiment. The experimental protocol was approved by the University of Minnesota's Institutional Review Board (Study IRB1402M47668). Sample size was informed by an a priori power analysis based on previous ADLD participant data [22] yielding a minimum requirement of 9 participants to detect significant pre‐post treatment differences with *α* = 0.05, *β* = 0.8, and effect size = 0.75.

**TABLE 1:** Clinical characteristics of study participants.

Subject ID	Sex	Age (years)	Time since diagnosis (years)	Voice handicap index at baseline (0–120)	Perceived speech effort at baseline (1–10)	Presence of vocal tremor	Perceived VTS‐induced voice change at post‐set 2
S01	F	34	12	103	5	No	**Noticeable**
S02	F	70	50	97	8.5	Yes	**Very noticeable**
S03	F	52	1.2	61	3	No	Unnoticeable
S04	F	49	10	57	8	No	Neutral
S05	F	59	11	112	9	No	**Noticeable**
S06	F	49	18	74	9	No	**Noticeable**
S07	M	49	9	69	9	No	Very unnoticeable
S08	F	66	23	70	4.5	No	**Very noticeable**
S09	F	60	8	62	3.5	Yes	**Noticeable**
S10	F	69	8.7	64	5	No	**Noticeable**
S11	F	55	9	99	7	No	Unnoticeable

*Note*: All participants were diagnosed with ABLD. Gender: F, female; M, male. Presence of voice tremor was diagnosed clinically. VHI score range is 0–120 (61–120 = severe severity). Perceived speech effort during speaking of unvoiced sentences at baseline represents the mean of two assessments prior to receiving VTS. Besides S10, no participant received regular Botulinum neurotoxin injections. Values in bold indicate noticeable to very noticeable change in perceived voice change.

### 2.2 Apparatus

#### 2.2.1 Wearable Device

**FIGURE 1:**
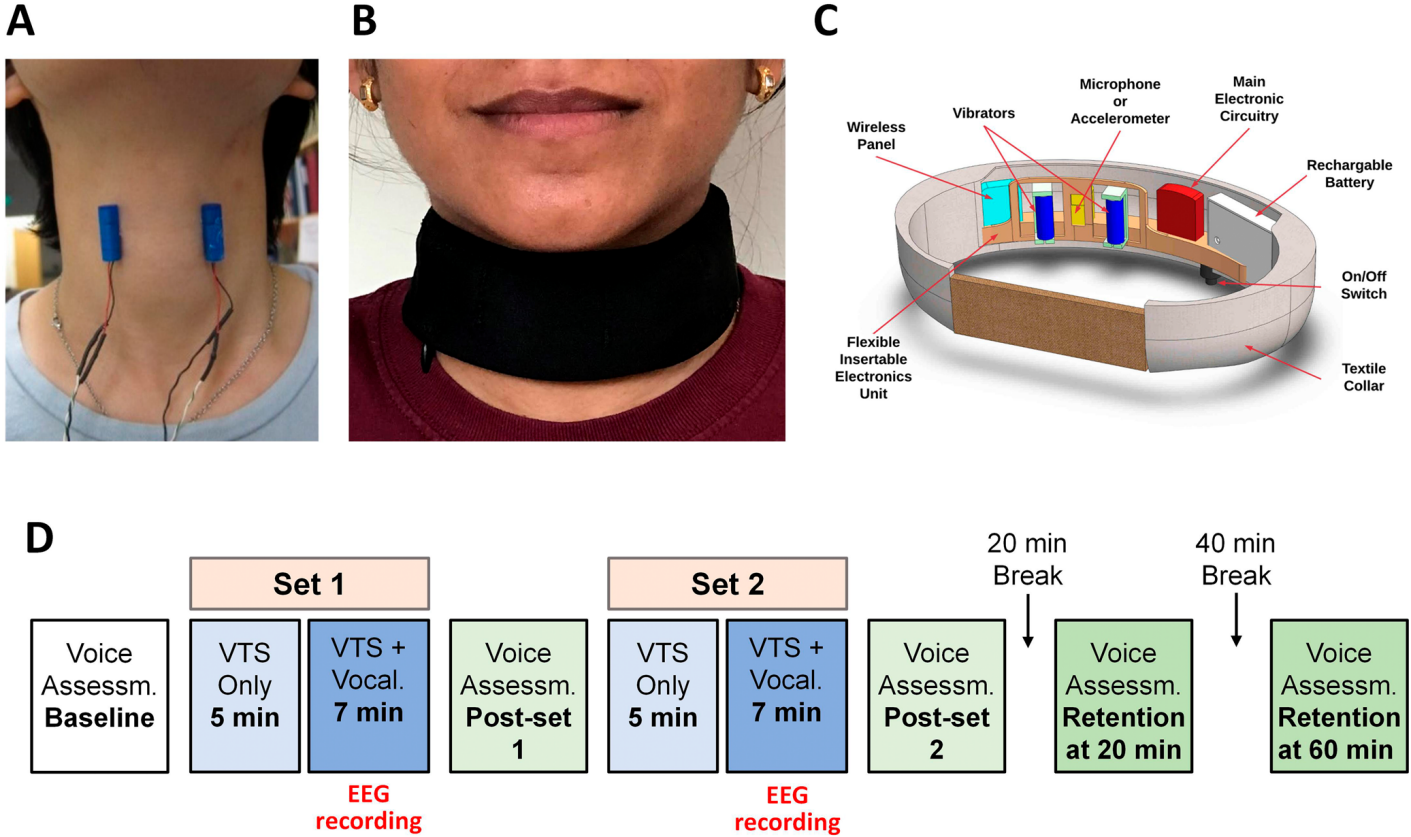
Vibration device and experimental protocol. (A) Position of the encapsulated vibrators. This photo shows the approximate anatomical placement of the vibrators above the skin of the voice box. (B) Placement of the wearable collar around the human neck. (C) The wearable collar that houses the vibrators shown in A, plus the control electronics and the power source. A microphone embedded in the collar recorded a participant's voice. Onset of vocalizing the vowel /a/ triggered the start of the vibrators. (D) Timeline of the experimental protocol. Participants received two sets of VTS for a total duration of 24 min. A total of five voice assessments were conducted, in which participants spoke the voiced/unvoiced test sentences, the unvoiced test words, and vocalized the vowels /a/ and /i/. [Color figure can be viewed in the online issue, which is available at www.laryngoscope.com]

A non‐invasive, collar‐like, wearable device applied VTS via two vibrators to the skin above the larynx (see Figure 1A for position of vibrators, Figure 1B for the device). The device incorporated speech‐detection technology activating the vibration during speaking or vocalization. The system (see Figure 1C) consisted of (1) two encapsulated vibrators (Model 307–100, Precision Microdrives Ltd., UK; 9 mm diameter, 25 mm length), (2) a flexible printed circuit board conforming to the neck, (3) a soft, wear‐resistive, and washable textile collar housing the vibrators, electronics, and power source, and (4) control software. Detailed technical specifications have been described previously [24].

#### 2.2.2 Voice and EEG Recording

All speech and voice signals were recorded using an external microphone (Sony ECM‐88B) located close to the participant's mouth and connected to an audio recorder (MixPre‐3 II, Sounds Devices LLC). EEG data were recorded with the ActiveTwo data acquisition system (Biosemi B.V., Netherlands) at the sampling rate of 4096 Hz using a 64‐channel EEG cap with an equiradial electrode placement. A 250‐ms, 1500 Hz auditory cue generated by RPvdsEx software (Tucker‐Davis Technologies Ltd., USA) guided participants through the experiment.

### 2.3 Experimental Procedure

The experiment took place in an electrically and acoustically shielded chamber at the Multisensory Perception Laboratory at the University of Minnesota. Participants sat comfortably, minimizing movement and focusing on a visual fixation point. For each participant, vibrator locations were adjusted to be positioned at the left and right edge of the thyroid cartilage (see Figure 1A).

#### 2.3.1 Experimental Protocol

The protocol comprised two VTS application sets (12 min each) and five voice assessment blocks (see Figure 1D). Each VTS set included two conditions: (1) Laryngeal vibration only (VTS Only) – 5 min of continuous laryngeal vibrators, and (2) participants vocalizing while laryngeal VTS was applied (*Vocalization+VTS*). VTS was applied at 100 Hz and a vibration amplitude of ~1.7 G (1 G = 9.81 m/s [2]). Preliminary work in our laboratory with healthy human volunteers showed that 100 Hz vibration generates peaks in the power spectrum of the voice signal within the frequency range known to stimulate laryngeal mechanoreceptors [25] and slowly adapting tactile mechanoreceptors. During the *Vocalization+VTS* condition, participants vocalized the vowel /a/ following an auditory cue. The collar triggered VTS activation after a 2200 ms delay, lasting for 2 s (i.e., VTS from 2200 to 4200 ms). Participants stopped vocalization after VTS was terminated. This was repeated 50 times with 4‐s rest intervals between trials.

Voice assessments occurred at baseline, after each VTS set, and at 20 and 60 min post‐VTS. Assessments included reading aloud standardized sentences devised for the evaluation of voice quality in AB and ADLD [26], vowel pronunciation (/i/ and /a/, 5 repetitions, 4 s each), and self‐rating of vocalization effort (scale: 0–10, no effort to maximal effort). (see Figure 1D).

### 2.4 EEG Signal Processing

EEG data were processed using the EEGLab toolbox and its plugin packages in MATLAB (MathWorks, MA, USA) with the aim to yield high‐quality, artifact‐free EEG data for analyzing cortical activity associated with vocalization events [27]. The processing pipeline began with the raw data being high‐pass filtered at 0.1 Hz using a zero‐phase FIR filter to address baseline drifts and low‐pass filtered at 50 Hz to remove high‐frequency noise. Power line noise and its harmonics were effectively eliminated using the *cleanline* plugin, which applies a zero‐phase Slepian window filter to target not only the fundamental power line frequency (e.g., 60 Hz) but also its lower and higher harmonics, ensuring a thorough removal of line noise artifacts. Time correction was performed by manually measuring vocalization onset and duration using audio recordings, and epochs were extracted from 1000 ms pre‐vocalization to 4000 ms post‐vocalization onset. The 1000 ms period prior to vocalization was used as the baseline (resting state) for cortical activity. The data were then re‐referenced to the common average of all electrodes to reduce the influence of non‐cortical sources, such as muscle or eye movements, and resampled to 500 Hz to balance computational effic**i**ency and signal fidelity. Independent Component Analysis (ICA) was performed on all channels using the *runica* algorithm to decompose the EEG signal into statistically independent sources. Automatic artifact rejection was then applied to the resulting components using the *SASICA* algorithm, which employs spatiotemporal criteria to identify and remove contaminated ICs, such as those related to muscle artifacts or eye movements. This step was critically important for ensuring the removal of artifacts that may have contaminated the EEG data during vowel vocalization. Finally, the remaining independent components were linearly summed to reconstruct the clean EEG signal, and the output dataset was used to extract EEG outcome measures.

### 2.5 Outcome Measures

#### 2.5.1 Objective Measures of Speech Quality

Speech recordings were analyzed using PRAAT software [28]. The speech signals from the voice assessments were broken into voiced sentences (VS), unvoiced sentences (UVS) and unvoiced words (UVW), with each assessment block consisting of 10 VS, 10 UVS, and 47 UNW. We determined the cumulative sentence duration (CSD) for voiced and UVS and the cumulative word duration (CWD) for UVW at baseline after each VTS set, and at 20 and 60 min post‐VTS (see Figure 1D). In addition, *smoothed cepstral peak prominence* (CPPS) was calculated as a measure of speech quality. CPPS is based on the acoustic signal's power spectrum and is defined as the difference in amplitude between the cepstral peak and the corresponding value on the regression line that is directly below the peak. Its unit is dB. CPPS correlates strongly with the severity of LD voice symptoms [29]. Improvements in voice quality are associated with an increase in CPPS. Relative changes in CWD and CSD were computed between baseline and the post‐intervention time points.

Improved voice symptoms could entail an increase in CPPS and/or a reduction in CSD or CWD. To quantify the clinical relevance of VTS‐induced changes, we established an increase of > 1 dB for CPPS and a reduction of > 2 s (representing 5% for CWD, 4% for unvoiced CSD, and 8% for voiced CSD) as minimal clinically meaningful improvements.

#### 2.5.2 Clinical Measures of Voice Symptom Severity

To evaluate the severity of the voice disorder, two measures were incorporated: the *Consensus Auditory‐Perceptual Evaluation of Voice* (CAPE‐V) inventory (scale of 0–100 with 100 indicating severe dysphonia) [30] and the *Voice Handicap Index* (VHI) [31]. A speech‐language pathologist with over 10 years of clinical voice experience, who was not involved in data collection and who was blinded with respect to the timepoint of the audio record, independently determined overall disease severity using CAPE‐V (score between 30 and 65 = moderate symptoms, 65–100 = severe symptoms). VHI is a self‐rated 30‐item inventory to indicate the impact of experienced voice problems or a voice disorder (score range: 0–120, score between 0 and 30 = mild severity; 31–60 = moderate severity; 61–120 = severe severity).

#### 2.5.3 Subjective Measures of Speech Quality

At the end of Set 2 of VTS, participants ranked their perceived changes in voice quality and effort when speaking the test sentences on a 5‐point scale (from 1 = *Very Unnoticeable* to 5 = *Very Noticeable*).

#### 2.5.4 Electrocortical Measures

The primary EEG measure was the event‐related spectral perturbation (ERSP), which calculates the mean event‐related deviation in spectral power relative to the baseline. ERSP was extracted for physiologically relevant frequency bands: theta (4–8 Hz), alpha (8–13 Hz), beta (13–30 Hz), using electrodes over laryngeal somatosensory–motor cortex in response to VTS. Neural oscillations in these bands, when detected over sensorimotor cortex, are known to be associated with voluntary movement preparation, execution, and somatosensory processing [32, 33]. Specifically, six electrode sites were analyzed: CP5 and CP6 (left and right somatosensory cortices), C5 and C6 (bilateral primary motor cortices), and FC5 and FC6 (premotor cortical areas).

The ERSP measurements were derived from the Vocalization+VTS trials to investigate the cortical response to VTS. Each 4000 ms trial was divided into two segments: (1) *Vocalization Only* before VTS onset (VTS‐off) and *Vocalization with Vibration* (VTS‐on). The ERSP was calculated by averaging 50 recorded epochs for each participant, separately for VTS‐off and VTS‐on segments. Time normalization was applied to the ERSP outcome to account for within‐and between‐participant variability in vocalization onset times and duration.

Changes in electrocortical activity in response to laryngeal VTS were quantified as the absolute ERSP changes for each band as:

(1) $$\text{Absolute change}:\Delta \text{ERSP}={\text{ERSP}}_{\mathrm{VTS}-\mathrm{on}}—{\text{ERSP}}_{\mathrm{VTS}-\mathrm{off}}\;\left[\mathrm{dB}\right]$$

Negative values of ΔERSP indicate a VTS‐induced event‐related desynchronization (ERD) in a respective cortical area that occurs in addition to the expected ERD due to motor activity, while positive values suggest a VTS‐induced event‐related synchronization (ERS).

## 3 Results

### 3.1 Acute Effects of VTS on Voice Symptoms

The application of VTS using the wearable collar proved to be feasible and safe. No adverse events were reported. At Baseline (Figure 1D), group median VHI was 70 (range: 57–112) with 10/11 participants exhibiting severe symptoms (> 61). Prior to receiving VTS, median perceived speech effort while speaking the UVS that are most sensitive to ABLD symptoms was 7 (range: 3–9) on a 10‐point scale (see Table 1). At post‐set 2 (i.e., after receiving 24 min VTS; Figure 1D), 7 out of 11 participants (64%) self‐reported a *noticeable* or *very noticeable* change in their voice symptoms (see Table 1).

**FIGURE 2:**
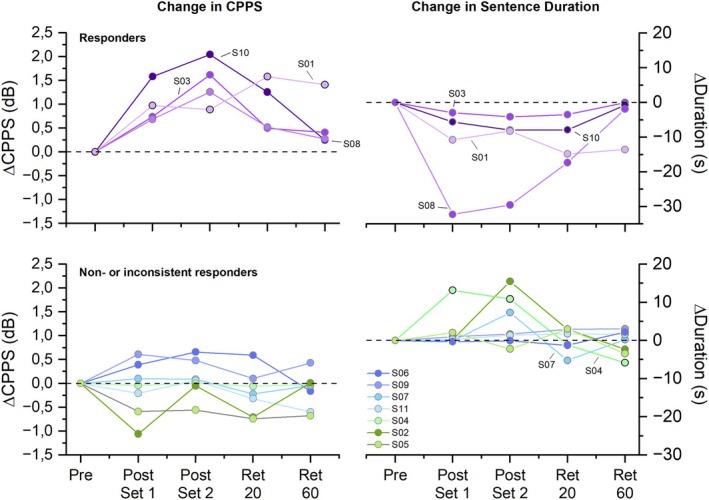
VTS‐related changes in voice outcome measures. Left panels depict change in CPPS relative to baseline (Pre) during speaking of unvoiced sentences, where an increase in dB indicates an improved voice. Right panels show the change in cumulative duration of unvoiced sentences, where a decrease in sentence duration corresponds to an improved voice. The criterion for classification as a responder was a VTS‐induced increase of at least 1 dB and/or a decrease of at least 2 s for cumulative sentence duration at two of the four timepoints. Each data point represents the performance of an individual participant at a respective time point: Post Set 1 = after 12 min VTS; Post Set 2 = after 24 min VTS; Ret 20 = 20 min after cessation of VTS; Ret 60 = 60 min after cessation of VTS. [Color figure can be viewed in the online issue, which is available at www.laryngoscope.com]

To determine the effect of VTS on objective measures of voice and speech, we analyzed the immediate and retained VTS‐induced change in CPPS, and the sentence and word duration variables CSD and CWD for each participant. The respective analysis revealed that 4/11 participants (36%) showed a consistent response to VTS for at least two time points in at least one of these voice measures (see Figure 2). To put the reported change in CPPS in context, mean CPPS at baseline was 7.06 dB (range: 5.16–11.47 dB). For the duration of the 47 unvoiced words, the same participants (S01, S03, S08, and S10) who exhibited reductions in CSD also showed reductions in CWD (mean: −8.0 s; range: 2.1–18.1 s). Twenty minutes after the application of VTS, 5/11 (45%) of participants exhibited reductions of > 2 s in CSD and/or CPPS > 1 dB (see Figure 2), an effect that was still observed in 3/11 (27%) of participants at the 60‐min retention time point. The related changes in CAPE‐V severity scores for the post set and retention time points are shown in Table 2.

**TABLE 2:** CAPE‐V overall severity score for unvoiced sentences of the two baseline assessments (0–100; 100 = most severe) for the different timepoints from Baseline to 60 min past the last VTS application.

Subject ID	CAPE‐V	CAPE‐V	CAPE‐V	CAPE‐V	CAPE‐V
	Baseline	Post set 1	Post set 2	Retention 20	Retention 60
	Prior to VTS	After 12 min VTS	After 24 min VTS	20 min past VTS	60 min past VTS
S01	**47**	52	46	37	35
S02	**32**	40	52	45	64
S03	**26**	25	22	23	21
S04	**85**	81	79	93	94
S05	**54**	61	58	57	63
S06	**24**	28	23	28	34
S07	**67**	71	75	79	74
S08	**78**	17	15	68	62
S09	**23**	19	21	25	22
S10	**53**	38	25	36	58
S11	**48**	62	47	61	55

*Note*: Bold values indicate the CAPE‐V rating at baseline prior to receiving VTS.

### 3.2 Acute Effects of VTS on Electrocortical Activity Over Somatosensory‐Motor Cortex

**FIGURE 3:**
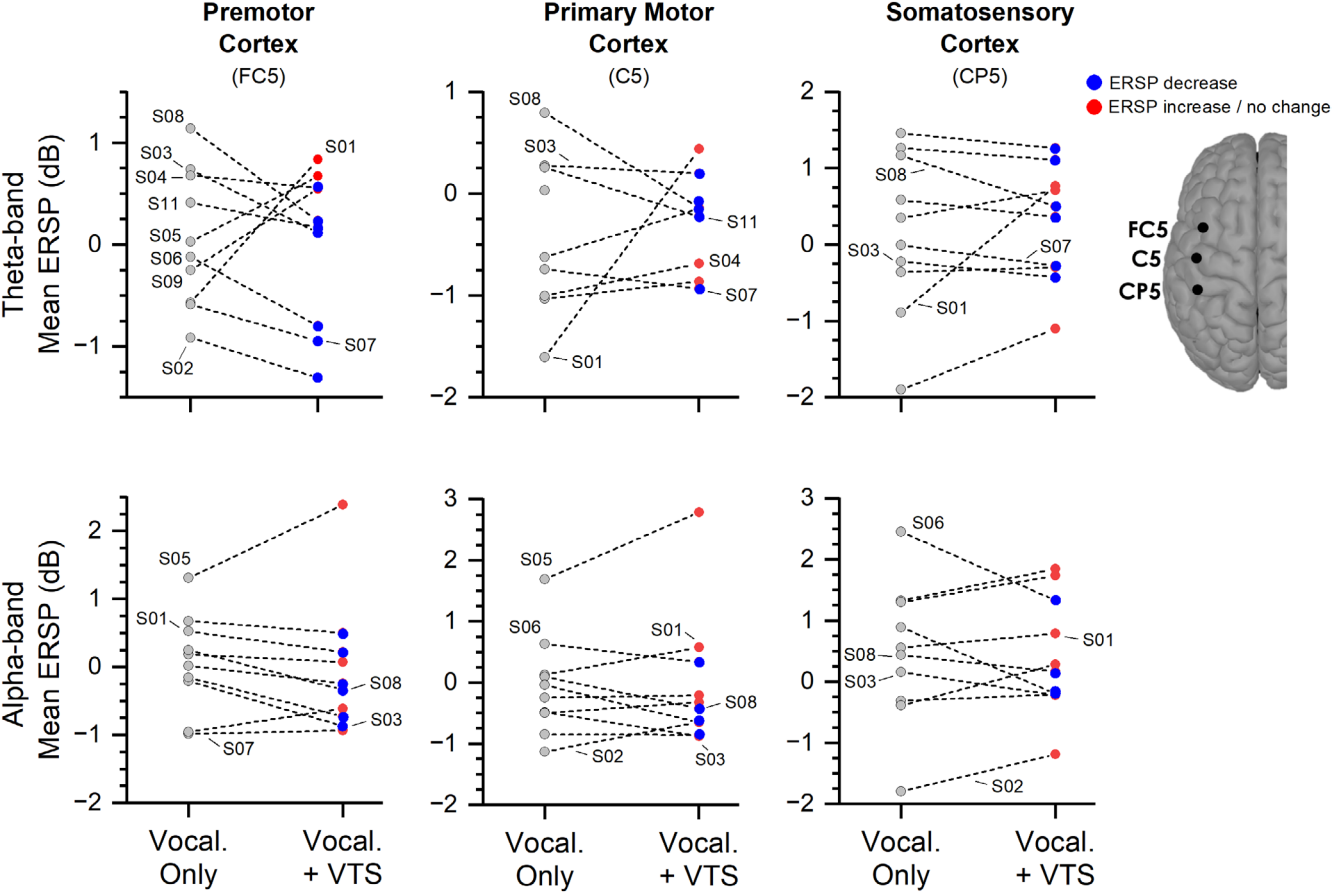
VTS‐related change in ERSP over left sensorimotor cortex after 24 min of VTS (Set 2). Each data point represents mean ERSP of a single participant for theta‐ and alpha‐band during the *Vocalization Only* or *Vocalization+VTS* periods. A VTS‐induced desynchronization in both theta and alpha bands was most consistently observed over premotor cortex (electrode FC5). [Color figure can be viewed in the online issue, which is available at www.laryngoscope.com]

**FIGURE 4:**
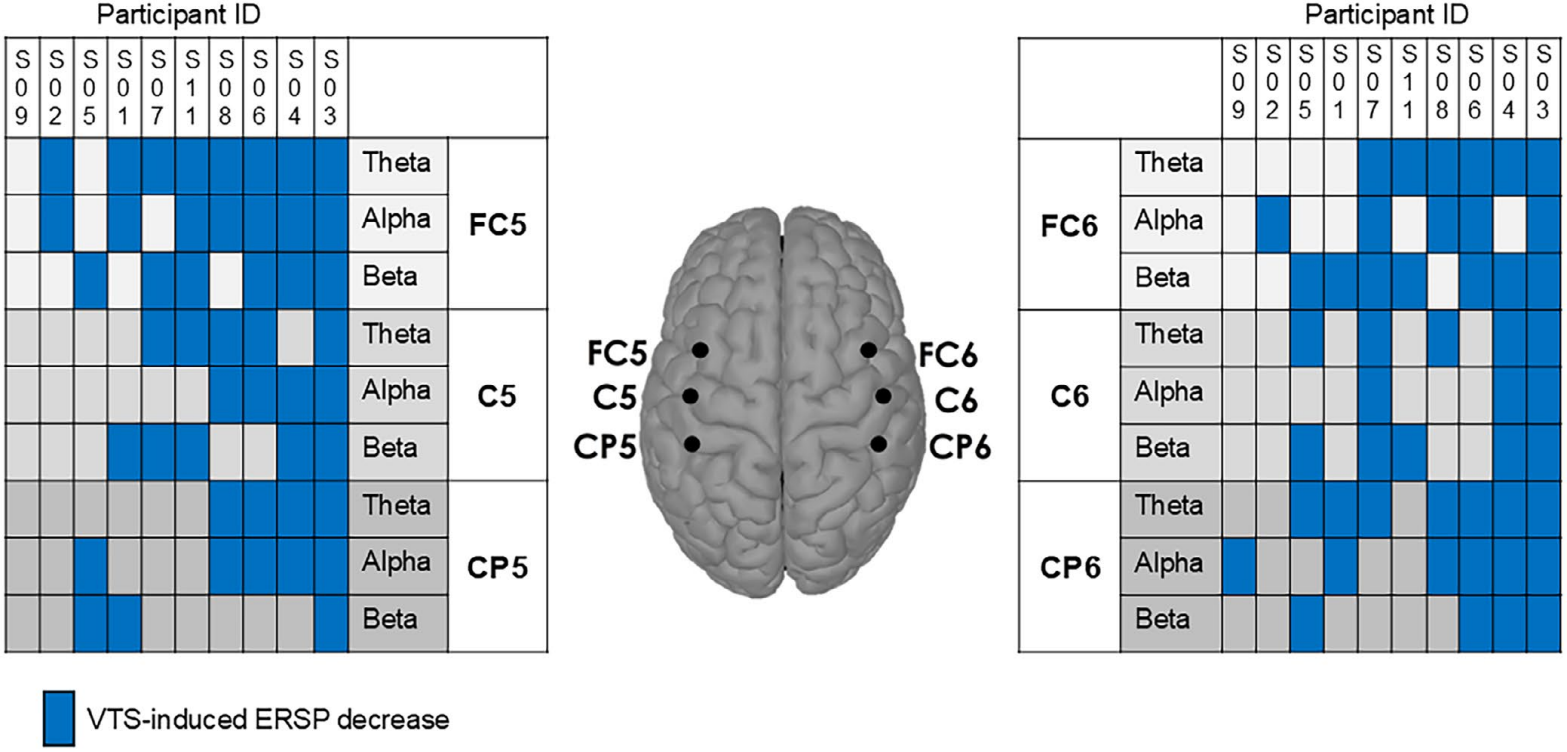
Summary of VTS‐induced desynchronization over the left and right sensorimotor cortices for theta, alpha, and beta‐band ERSP. Participants are sorted from right to left according to the consistency of the electrocortical response to VTS over the left sensorimotor cortex. [Color figure can be viewed in the online issue, which is available at www.laryngoscope.com]

The EEG signal analysis was based on the data of 10 participants (S10 recordings were unusable). VTS induced an ERD over the left and/or right laryngeal somatosensory‐motor cortex in 9/10 participants. Consistency of ERD across participants was most pronounced in the theta band, but also visible in the alpha and beta bands. With respect to the theta band, an ERD was observable in 8/10 (80%) participants over somatosensory, primary motor, and premotor cortical areas. The individual participant theta and alpha band ERSP data are shown in Figure 3. For each participant, the VTS‐induced absolute change in ERSP over the left somatosensory‐motor cortex relative to the *Vocalization Only* period is shown in Table 3 for all three frequency bands. Analysis of the right‐hemisphere ERSP data indicates that a VTS‐induced ERD across the three bands was also present over the right somatosensory‐motor cortex (see Figure 4).

**TABLE 3:** VTS‐induced absolute change in ERSP over left somatosensory‐motor cortex for each participant at set 2.

	Premotor cortex (FC5)			Motor cortex (C5)			Somatosensory cortex (CP5)		
	Theta	Alpha	Beta	Theta	Alpha	Beta	Theta	Alpha	Beta
S01	1.41	**−0.26**	**−0.04**	2.04	0.44	**−0.12**	1.66	0.23	**−0.15**
S02	**−0.39**	**−0.11**	0.45	0.17	0.49	1.17	0.80	0.61	1.50
S03	**−0.62**	**−0.66**	**−0.63**	**−0.08**	**−0.39**	**−0.13**	**−0.21**	**−0.38**	**−0.49**
S04	**−0.12**	**−0.18**	**−0.33**	0.32	0.04	**−0.41**	**−0.22**	−1.08	0.18
S05	0.64	1.08	**−0.46**	0.36	1.10	**−0.70**	**−0.19**	0.52	**−0.94**
S06	**−0.68**	**−0.31**	**−0.35**	−0.10	−0.30	0.09	**−0.16**	**−1.13**	**−0.29**
S07	**−0.36**	0.05	**−0.32**	−0.19	0.18	0.16	**−0.27**	0.44	0.64
S08	**−0.91**	**−0.58**	0.44	−0.93	−0.52	0.40	**−0.67**	**−0.27**	0.44
S09	0.80	0.34	0.35	0.48	−0.01	0.35	0.06	0.10	0.29
S11	**−0.24**	**−0.57**	**−0.19**	**−0.48**	**−0.61**	**−0.04**	0.37	0.67	**−0.12**

*Note*: Values are in dB. Negative values (bold font) indicate an event‐related depression in a particular band or electrode. Note that ERD over premotor cortex was observed in at least one band in all but one participant (SD09).

Relating observed VTS‐induced ERD in the three frequency bands to changes in objective voice measures showed that 4/10 participants (S01, S03, S05, and S08) identified as responders based on objective voice measures (see Figure 2) and 7/10 participants who reported subjective improvement in voice quality exhibited VTS‐related ERD over left and/or right somatosensory‐motor cortex in one or more frequency bands. Note that 3/10 participants (S04, S07, and S11) who exhibited VTS‐induced desynchronization over one or both brain hemispheres in one or more frequency bands did not show marked improvements in any objective or subjective voice measure (see Figure 4).

## 4 Discussion

This study sought to determine whether laryngeal VTS can reduce voice symptoms in people with abductor‐type LD. Our previous work had documented that, depending on the outcome measure, laryngeal VTS induced meaningful acute changes in speech effort and voice quality in up to 69% of people with adductor‐type LD [22, 23]. However, no evidence existed if people with ABLD would respond to VTS. The main findings of the current study are as follows: First, 64% of participants reported a *noticeable* or *very noticeable* change in their voice symptoms after VTS application. Second, analysis of objective voice measures such as CPPS and CSD revealed measurable improvements in voice quality in 45% of participants 20 min past the cessation of VTS. Third, VTS induced a desynchronization of neuronal firing patterns over left and right somatosensory‐motor cortical areas that was most prominent over left premotor cortex.

The results indicate that VTS can be safely applied to people with ABLD using a wearable collar with embedded vibrators [24]. VTS as an intervention acts fast. The effects on voice symptoms were observable after a 12‐min application of VTS and most pronounced after 24 min (see Figure 2). After receiving VTS, the subjective impression of most participants (7/11) was positive (*noticeable* or *very noticeable* change). A corresponding analysis of the objective voice measures such as CPPS and duration of UVS that are known to be sensitive to ABLD voice symptoms partially corroborated the subjective impression, with up to 45% of participants showing a meaningful improvement in at least one voice measure immediately after or 20 min past VTS. With respect to the retention of a treatment effect, the data indicate that it decayed quickly and was no longer observable after 60 min (see Figure 2). These findings broadly align with the empirical evidence from previous research on ADLD, where a single 30‐min VTS session induced acute improvement in speech quality in 69% of the participants [22], and an 11‐week longitudinal study showed that 57% experienced repeated meaningful benefits such as reduced voice effort [23].

Our EEG analysis focused on low frequency bands (4–30 Hz), because cortical activity in these bands is associated with motor planning, control of muscle tone, and processing of somatosensory afferents. Previous research documented an excessive rise of premotor/motor cortical β‐oscillations during motor planning in cervical dystonia [34] and an abnormally high synchronous theta‐band activity within and across cortical neural networks involved in voice production in LD [12]. For people with ADLD, the electrocortical effect of VTS was a suppression of this excessive synchronization in theta‐band over the left somatosensory‐motor cortex. Such suppression of theta oscillations has been observed in people with cervical dystonia who apply effective sensory tricks, suggesting that VTS may activate a similar neurophysiological mechanism [22]. The EEG signal analysis of our sample indicates that the same mechanism is behind the effectiveness of VTS in ABLD, with the addition that VTS‐related synchronization was also visible in alpha‐ and beta‐band ERSP.

### 4.1 Limitations

This study is a proof‐of‐concept study that necessarily has limitations. It does not constitute a placebo‐controlled randomized trial with a sham group and a group of healthy controls. We did not employ a healthy control group because previous research had already demonstrated the electrocortical response of healthy controls to VTS [22, 23]. We did not employ a sham group. Thus, we cannot exclude the possibility of a placebo effect. However, given the rather immediate effect of VTS, knowing that the effect was shown to be repeatable over an 11‐week period in a randomized clinical trial [23], and having electrocortical data showing a very fast neural response to VTS, it is highly unlikely that a behavioral response to VTS can be solely understood as an unspecific placebo effect. One also needs to recognize the discrepancy between subjective and objective measures of speech, with almost two‐thirds of participants reporting improved ease of speech, while objective voice changes were observed in less than half of our sample.

## 5 Conclusion

Applying laryngeal VTS proved to be feasible and safe. This proof‐of‐concept study demonstrated that laryngeal VTS can lead to temporary symptom relief in people with ABLD. Given that the therapeutic arsenal for treating ABLD is limited, VTS may provide an additional treatment avenue to obtain temporary symptom relief. The findings align with previous reports on the effectiveness of VTS in treating voice symptoms in ADLD.

## Conflicts of Interest

The authors declare no conflicts of interest.
